# Development of Molecular Markers for Bacterial Leaf Streak Resistance Gene *bls2* and Breeding of New Resistance Lines in Rice

**DOI:** 10.3390/ijms26115264

**Published:** 2025-05-30

**Authors:** Jieyi Huang, Xuan Wei, Min Tang, Ziqiu Deng, Yi Lan, Fang Liu

**Affiliations:** State Key Laboratory for Conservation and Utilization of Subtropical Agro-Bioresources, College of Agriculture, Guangxi University, Nanning 530004, China; 2217301013@st.gxu.edu.cn (J.H.); 2317301051@st.gxu.cn (X.W.); 2017301036@st.gxu.edu.cn (M.T.); 2117301005@st.gxu.edu.cn (Z.D.); 2031200139@st.gxu.edu.cn (Y.L.)

**Keywords:** rice, bacterial leaf streak, resistance improvement, molecular marker-assisted selection, agronomic traits

## Abstract

Bacterial leaf streak (BLS) is one of the internationally significant quarantine diseases in rice. Effectively utilizing BLS resistance genes from wild rice (*Oryza rufipogon* Griff.) to breed new varieties offers a fundamental solution for BLS control. This study focused on the fine mapping of the BLS resistance gene *bls2* and the development of closely linked molecular markers for breeding BLS-resistant lines. Using a Guangxi common wild rice accession DY19 (carrying *bls2*) as the donor parent and the highly BLS-susceptible indica rice variety 9311 as the recipient parent, BLS-resistant rice lines were developed through multiple generations of backcrossing and selfing, incorporating molecular marker-assisted selection (MAS), single nucleotide polymorphism(SNP) chip genotyping, pathogen inoculation assays, and agronomic trait evaluation. The results showed that *bls2* was delimited to a 113 kb interval between the molecular markers ID2 and ID5 on chromosome 2, with both markers exhibiting over 98% accuracy in detecting *bls2*. Four stable new lines carrying the *bls2* segment were obtained in the BC_5_F_4_ generation. These four lines showed highly significant differences in BLS resistance compared with 9311, demonstrating moderate resistance or higher with average lesion lengths ranging from 0.69 to 1.26 cm. Importantly, no significant differences were observed between these resistant lines and 9311 in key agronomic traits, including plant height, number of effective panicles, panicle length, seed setting rate, grain length, grain width, length-to-width ratio, and 1000-grain weight. Collectively, two molecular markers closely linked to *bls2* were developed, which can be effectively applied in MAS, and four new lines with significantly enhanced resistance to BLS and excellent agronomic traits were obtained. These findings provide technical support and core germplasm resources for BLS resistance breeding.

## 1. Introduction

Rice (*Oryza sativa* L.) as one of the world’s major food crops, sustains nearly 60% of the global population. However, rice production faces multiple threats, including biotic and abiotic stresses. Among these, diseases caused by fungi, bacteria, nematodes, and viruses are one of the primary reasons for severe yield losses in rice, posing a significant threat to global food security [1]. Bacterial leaf streak (BLS), caused by the Gram-negative bacterium *Xanthomonas oryzae* pv. *oryzicola* (*Xoc*), is the fourth most destructive rice disease, following bacterial leaf blight, blast, and sheath blight. It is characterized by early infection in the growth cycle, rapid spread, high destructiveness, and frequent recurrence [2]. BLS is particularly prevalent in warm and humid regions, especially during rainy seasons or in areas with well-developed irrigation systems, where the disease spreads rapidly. Studies have shown that BLS can reduce rice yields by 6–40%, with losses exceeding 40% in severely affected regions. Currently, registered chemical agents for BLS control are limited and generally ineffective [3]. In practice, excessive pesticide application is common, leading to severe ecological pollution, increased toxic residues in rice, and significant threats to food production safety. Therefore, breeding resistant rice varieties using BLS resistance genes is considered the most effective, economical, and environmentally friendly method to prevent BLS outbreaks.

Rice resistance to BLS is mostly controlled by quantitative trait loci (QTLs), with a minority governed by major genes [4,5]. To date, only two dominant resistance genes (*Xo1* and *Xo2*), three recessive resistance genes (*qBlsr5a*, *bls1*, and *bls2*), and one non-host resistance gene (*Rxo1*) have been identified [6]. *Rxo1*, cloned from maize by Zhao et al. [7], triggers an immune response against *Xoc*. It encodes a protein with an NBS-LRR (nucleotide binding site–leucine rich repeat) domain. When introduced and expressed in a susceptible rice variety, this gene can confer resistance to *Xoc* [8]. Triplett et al. [9] identified a dominant major resistance gene, *Xo1*, within a 1.09 Mb region on the long arm of chromosome 4, which exhibits high resistance to African *Xoc* strains but not to Asian strains. Read et al. [10] cloned *Xo1*, which encodes a protein containing an NBS-LRR domain and activates the rice defense response by recognizing the transcription activator-like effector (TALE) secreted by *Xoc*. However, the resistance mediated by *Xo1* can be suppressed by iTALE (a TALE variant). Chen et al. [11] identified a dominant major gene, *Xo2*, from the Bangladeshi resource variety BHADOIA 303. The gene was mapped to a 110 kb region between RM12941 and D6-1 on chromosome 2. Xie et al. [12] preliminarily cloned *qBlsr5a*, which was found to be allelic to the bacterial blight resistance gene *xa5*. He et al. [13] mapped the resistance gene *bls1* from common wild rice DP3 to a 4 cM interval between RM587 and RM510 on chromosome 6. Subsequently, Ma et al. [14] successfully cloned this gene, which encodes a mitogen-activated protein kinase (OsMAPK6). Their studies demonstrated that low expression of *bls1* or overexpression of *BLS1* could enhance or reduce rice resistance to the specific *Xoc* strain JZ-8, respectively. Furthermore, both low expression of *bls1* and overexpression of *BLS1* could increase non-race-specific broad spectrum resistance in rice. The major recessive gene *bls2* was identified by Shi et al. [15] from Guangxi common wild rice DY19 and mapped to a 4 cM interval between molecular markers SL03 and SL04 on chromosome 2.

Currently, most of the identified BLS resistance genes are QTLs with minor phenotypic effects, making their cloning and utilization challenging. Although some major BLS resistance genes have been mapped and cloned, few have been successfully applied in breeding programs or incorporated into commercial varieties. Therefore, it is crucial to develop user-friendly molecular markers for resistance genes to facilitate their introgression into susceptible rice varieties. This will enable the creation of novel germplasm with BLS resistance, thereby accelerating the application of superior resistance genes in breeding practices.

This study aims to develop closely linked molecular markers for *bls2* and introgress this gene into the elite indica rice variety 9311 through multiple generations of backcrossing and selfing, combined with foreground/background selection using molecular markers and SNP(single nucleotide polymorphism) chip analysis, thereby developing rice lines resistant to BLS. Finally, these lines will be evaluated for BLS resistance using field needle-puncture inoculation methods while their key agronomic traits will be investigated to assess the breeding value of *bls2*. The results will provide new core germplasm materials for further breeding of BLS-resistant rice varieties.

## 2. Results

### 2.1. Fine Mapping of bls2

*bls2* was initially mapped to a 4 cM interval between markers SL03 and SL04 on chromosome 2 [15]. To fine-map *bls2*, we performed genotyping analysis of 1527 BC_5_F_2_ individuals and 529 BC_5_F_3_ individuals using these two markers, from which 189 recombinant plants were identified. Considering the relatively large physical distance between SL03 and SL04 and the substantial number of recombinant plants obtained, we conducted subsequent analysis using a subset of recombinant plants together with an additional marker RM13592. This enabled us to delimit *bls2* to the region between RM13592 and SL04, with a reference physical distance of approximately 490 kb. These two markers were then used to reanalyze the 189 recombinant plants obtained previously. As a result, 48 plants (2.33% of the total individuals) with recombination between RM13592 and SL04 were identified, which were advanced for seed propagation.

**Figure 1:**
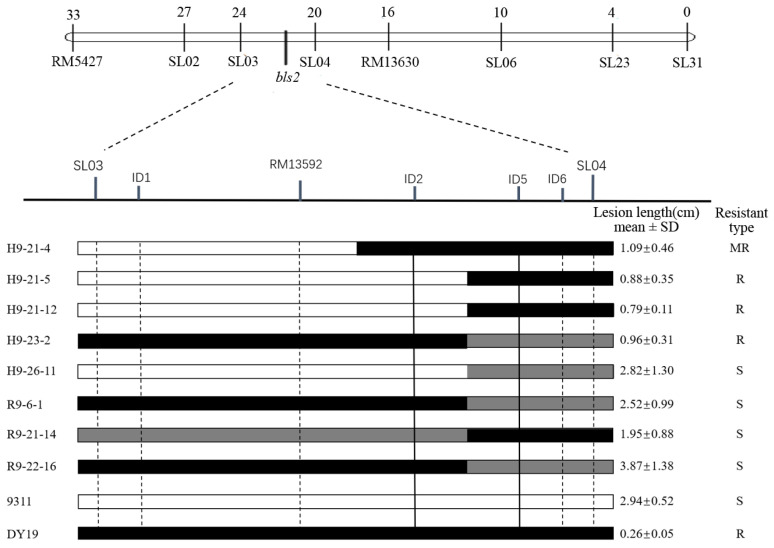
Fine mapping of *bls2*. The white, black and grey bars denoted the marker genotypes of ‘9311’, ‘DY19’ and heterozygote, respectively. R9-6-1, R9-21-14, R9-22-16 were individuals from the BC_5_F_2_ population and others from the BC_5_F_3_ population.

Within the target region, additional Indel (insertion-deletion) markers were designed and screened for polymorphism. Ultimately, seven polymorphic markers (SL03, ID1, RM13592, ID2, ID5, ID6, and SL04) between the parents were used to genotype the 48 recombinant plants. The BLS resistance phenotype of the progeny lines (BC_5_F_3_/BC_5_F_4_) derived from these 48 recombinant plants were evaluated using the needle-puncture inoculation method, with the mean lesion length of each line serving as the phenotypic value for the corresponding recombinant plant. The genotypes and phenotypic values of key recombinant plants are shown in Figure 1. By integrating phenotypic and genotypic data, *bls2* was localized to a 113 kb region between ID2 and ID5 (Figure 1). According to the Rice Annotation Project (https://rapdb.dna.affrc.go.jp/), there are seven candidate genes in this 113 kb region, among which *Os02g0614966*, *Os02g0615300*, *Os02g0615400*, *Os02g0615500*, and *Os02g0615800* are LRR (leucine-rich repeat)-containing protein genes (Table 1).

### 2.2. Accuracy of ID2 and ID5 Assisted Selection

**Figure 2:**
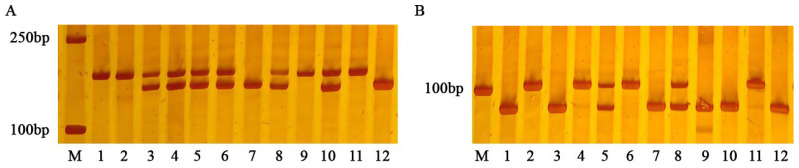
Polymorphism of molecular markers ID2 (**A**) and ID5 (**B**) in parents and BC_5_F_2_ population (**A**) M: DL2000 DNA Marker; 11: Susceptible parent 9311; 12: Resistant parent DY19; 1, 2, 9: Homozygous susceptible marker genotypes; 7: Homozygous resistant marker genotypes; 3, 4, 5, 6, 8, 10: Heterozygous marker genotypes. (**B**) M: DL2000 DNA Marker; 11: Susceptible parent 9311; 12: Resistant parent DY19; 2, 4, 6: Homozygous susceptible marker genotypes; 1, 3, 7, 9, 10: Homozygous resistant marker genotypes; 5, 8: Heterozygous marker genotypes.

Genotyping of randomly selected BC_5_F_2_ plants was performed using molecular markers ID2 and ID5 (Figure 2), and the lesion length of the plants was investigated. The consistency rate between marker genotypes and resistance phenotypes to BLS was calculated in Table 2.

Using molecular marker ID2, 151 resistant plants were detected, of which 149 exhibited the resistant marker genotype, resulting in a genotype-phenotype consistency rate of 98.68%. Among 156 susceptible plants tested, all 156 individuals showed the susceptible marker genotype, achieving a 100.00% consistency rate. Thus, the overall marker-assisted selection (MAS) accuracy of ID2 in the group was 99.35%.

For molecular marker ID5, 147 out of 151 resistant plants displayed the resistant genotype, yielding a consistency rate of 97.35%. All 156 susceptible plants tested carried the susceptible genotype, maintaining a 100.00% consistency rate. Consequently, the overall MAS accuracy of ID5 in the group was 98.70%.

When dual markers were applied, the genotype-phenotype consistency rate was 97.35% for resistant plants and 100.00% for susceptible plants, resulting in an overall accuracy of 98.70% for dual-marker-assisted selection.

These results demonstrate that both ID2 and ID5 exhibit high accuracy in selecting resistant and susceptible individuals within the rice population.

### 2.3. Development of New BLS-Resistant Lines

The plants in the BC_5_F_2_ population were screened using molecular markers ID2 and ID5, coupled with inoculation identification. Those exhibiting both the resistant marker genotype and the resistant phenotype were selected and subsequently self-pollinated in both early and late cropping seasons. Ultimately, four new lines Y9-1, Y9-2, Y9-3, and Y9-4 were selected from the BC_5_F_4_ generation, all carrying homozygous resistant marker genotypes and demonstrating stably enhanced resistance to BLS.

### 2.4. SNP Detection of the Resistant Lines

**Figure 3:**
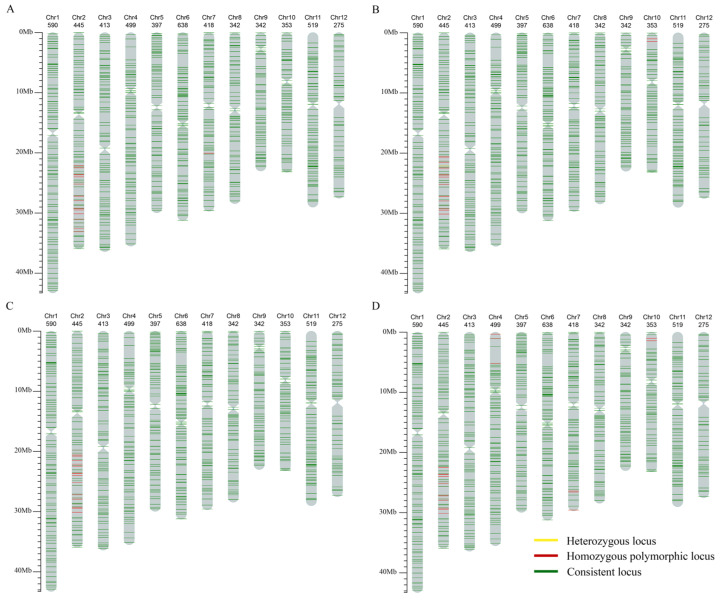
Detection results of 1 K mGPS SNP microarray of the resistance lines. (**A**) Y9-1. (**B**) Y9-2. (**C**) Y9-3. (**D**) Y9-4. The ordinate axis represents the physical position on chromosome. The numerical values above each chromosome represent the number of markers with homozygous genotypes in the recurrent parent 9311.

First, the resistant and susceptible parents were analyzed using the 1K mGPS SNP chip to screen for markers with homozygous and consistent genotypes in both parents, which were then used to analyze the resistant lines. The results showed that the genotypes of the four resistant lines were completely consistent with those of the parents at these markers, confirming that all four resistant lines were offspring of 9311 and DY19, with no cross-contamination or mixing of materials during the breeding process. Further, marks with polymorphic and homozygous genotypes between the two parents were selected for genotyping analysis of the resistant lines. A total of 5246 SNP markers were used for detection, of which 2569 markers were polymorphic between the two parents, accounting for 48.97% (Supplementary Table S1). A schematic diagram was generated to visualize the genotype comparison results with the recurrent parent 9311 (Figure 3). The results indicated that the differential loci on chromosome 2 between the four lines and 9311 were consistent with the previously mapped region of *bls2*. The marker consistency rates between Y9-1, Y9-2, Y9-3, Y9-4 and 9311 were 98.39%, 98.05%, 98.37% and 98.27%, respectively (Supplementary Table S1), indicating that the genetic backgrounds of the four resistant lines were highly similar to that of 9311.

### 2.5. Resistance Responses of the Resistant Lines to Different Xoc Strains

The four resistant lines exhibited varying levels of resistance to the tested *Xoc* strains at the same growth stage, ranked from strongest to weakest as follows: GX01, BNN3-*Xoc*, YN-*Xoc*, and 2019-*Xoc*. For the same *Xoc* strain, the resistance of the four lines across different growth stages followed this descending order were as follows: booting stage, seedling stage, and tillering stage. All four selected lines showed resistance against the four highly virulent strains (GX01, BNN3-*Xoc*, 2019-*Xoc*, YN-*Xoc*) during the seedling, tillering, and booting stages, while 9311 was susceptible to all four *Xoc* strains at every growth stage (Table 3). These results indicate that *bls2* confers BLS resistance against four *Xoc* strains throughout the entire growth period.

### 2.6. Agronomic Traits of the Resistant Lines

**Figure 4:**
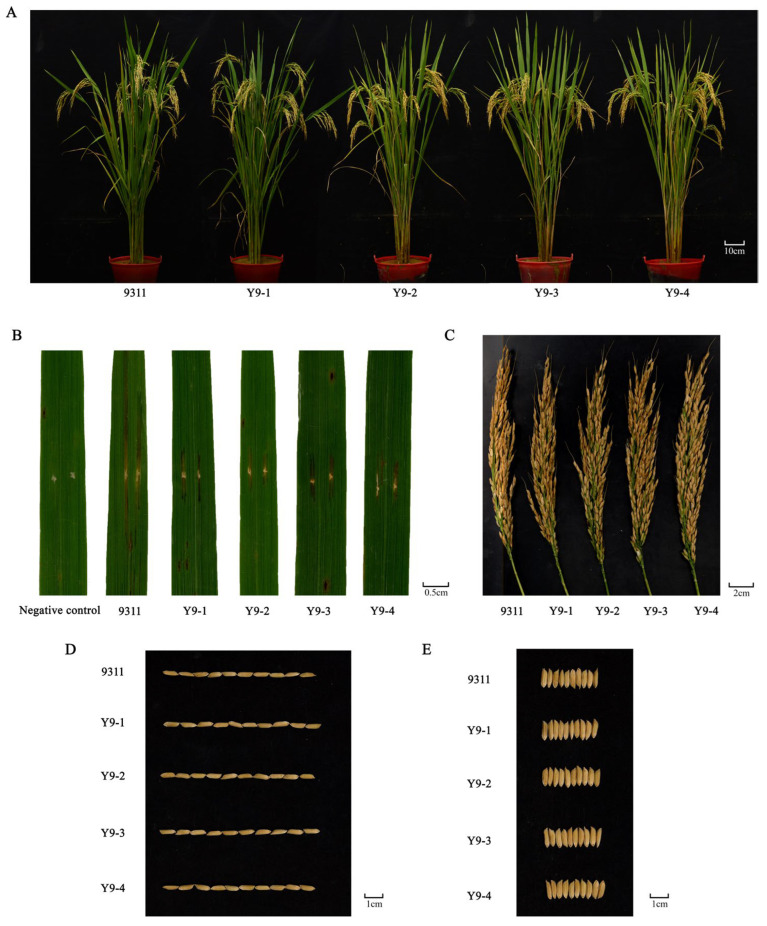
Phenotypic comparison of the resistant lines with parent 9311. (**A**) Plant phenotype. (**B**) Leaf lesions. (**C**) Spike phenotype. (**D**) Grain length; (**E**) Grain width. The *Xoc* strain used in (**B**) is GX01.

The four resistant lines exhibited significantly shorter lesion lengths than 9311 following GX01 inoculation at the tillering stage (Figure 4B). In terms of agronomic traits, there were no significant differences between the resistant strains Y9-1, Y9-2, Y9-3, Y9-4 and 9311 in plant height, number of effective panicles, panicle length, seed setting rate, grain length, grain width, length-to-width ratio, and 1000-grain weight (Figure 4A,C–E and Table 4).

## 3. Discussion

### 3.1. The Candidate Genes of bls2

In this study, the BLS resistance gene *bls2* from Guangxi common wild rice was mapped within a 113 kb region between molecular markers ID2 and ID5 on chromosome 2 using map-based cloning. There are seven annotated genes in this 113 kb region, including five LRR-containing protein genes. *Os02g0614966* and *Os02g0615400* encode LRR domain containing protein. *Os02g0615300*, *Os02g0615500* and *Os02g0615800* encode LRR-receptor-like kinase (LRR-RLK). Among them, *Os02g0615800,* named *LP2*, was reported to serve as a negative regulator in drought response. The expression of *LP2* was down-regulated by drought and abscisic acid (ABA). Transgenic plants overexpressing *LP2* accumulated less H_2_O_2_, had more open stomata in leaves, and showed hypersensitivity to drought stress [16]. However, whether the function of this gene is involved in disease resistance remains unclear.

LRR-containing proteins have been reported to play an important role in plant defense responses against pathogens. For instance, *SlLRP* in tomato [17] and *NtLRP1* in tobacco [18] are both responsive to pathogen infection. In rice, a simple extracellular LRR domain gene *OsLRR1*, whose transcription levels are induced by pathogen infection, wounding and treatment with salicylic acid (SA) or jasmonic acid (JA). OsLRR1 enters the endosomal pathway and interacts with the plasma membrane-localized OsHIR1. Heterologous expression of OsLRR1 in Arabidopsis enhances resistance to *Pseudomonas syringae* by upregulating the transcription of defense-related genes associated with the SA and JA pathways [19]. Additionally, OsLRR1 is required for the defense response mediated by the bacterial blight resistance gene *Xa21* against *Xanthomonas oryzae* pv. *Oryzae*(*Xoo*) [20]. *Xa21*, the first cloned resistance gene in rice [21], encodes a receptor-like kinase with three critical domains: an extracellular LRR region serving as the receptor, a transmembrane domain, and an intracellular Ser/Thr kinase domain. The extracellular LRR region recognizes and binds the *Xoo* effector protein RaxX, thereby initiating the PTI (Pathogen associated molecular pattern-triggered immunity) process [22,23]. In addition to *Xa21*, the other cloned bacterial blight resistance genes *Xa3/Xa26*, and *Xa14* also encode LRR- RLKs, While *Xa1*, *Xa2*, *Xa14*, *Xa31* and *Xa47* encode NBS-LRR proteins [24].

Resistance genes encoding LRR-RLKs and NBS-LRR proteins are often clustered in the rice genome and form the genetic foundation of qualitative resistance, allowing rice plants to effectively defend against pathogens [25]. Therefore, it appears that these five LRR-containing protein genes are more possibly related to BLS resistance. However, this prediction is only a preliminary speculation, and further functional verification of the target genes is underway.

### 3.2. Development of bls2 Molecular Marker

MAS utilizes markers tightly linked to target genes to detect and select genotypes, offering higher efficiency than conventional breeding techniques and facilitating the pyramiding of resistance genes [26]. The efficacy of MAS depends on marker-gene linkage strength, polymorphism reliability and technical reproducibility. Low marker accuracy or insufficient markers will increase the workload of phenotypic identification during the breeding process and may even lead to the loss of resistance genes.

The application of MAS in resistance breeding relies on the identification of resistance genes. Currently, there are still relatively few reports on the mapping and cloning of major genes conferring resistance to BLS. He et al. [13] and Ma et al. [14] identified and cloned a recessive gene, *bls1*, located between RM587 and RM510 on chromosome 6 from Guangxi common wild rice accession DP3, which encodes a mitogen-activated protein kinase (OsMAPK6). While another recessive gene *bls2* was preliminarily mapped within a 4 cM interval between SL03 and SL04 on chromosome 2 [15]. To narrow down the *bls2* localization region, this study performed fine mapping using BC_5_F_2_ and BC_5_F_3_ population, with additional polymorphic markers developed within the target region. *bls2* was ultimately delimited to a 113 kb interval between molecular markers ID2 and ID5 on chromosome 2, laying a solid foundation for its cloning and functional studies. Both *bls1* and *bls2* originate from Guangxi common wild rice, demonstrating the feasibility of mining major BLS resistance genes from this resource.

To enable precise selection of *bls2* and rapidly apply it to the improvement of BLS resistance in cultivated rice varieties, the MAS accuracy of its linked markers was evaluated. By genotyping and phenotyping 151 resistant plants and 156 susceptible plants, it was found that the accuracy rates of ID2 and ID5 in detecting *bls2* exceeded 98%, which meets the requirements for MAS breeding (with an accuracy threshold of over 80%). Both ID2 and ID5 exhibited excellent reproducibility, polymorphism, PCR amplification efficiency, and genotyping clarity, making them robust tools for MAS in breeding rice varieties resistant to BLS.

### 3.3. Application of MAS in the Development of New Resistant Lines

Hybrid breeding involves crossing two parental materials to exploit recombination of favorable genes, thereby creating new genetic variations. Based on breeding objectives, new varieties combining the advantages of both parents are developed by screening and breeding from the offspring [27]. While hybrid breeding produces offspring with rich genetic backgrounds and a high likelihood of desirable traits, it suffers from long breeding cycles and low efficiency. MAS shortens the breeding duration, accelerates the breeding process, improves selection efficiency, and overcomes the limitations of conventional breeding methods [28].

In traditional breeding, constructing near-isogenic lines (NILs) with high background recovery rates requires at least four to five backcrossing generations. However, for NILs carrying homozygous recessive genes, eight to ten generations of backcrossing and selfing are typically recommended [18]. Tanksley and McCouch [29] demonstrated that using MAS, a high background recovery rate can be achieved after just three backcrosses. Ellur et al. [30] and Sagar et al. [31] showed that by combining strict phenotypic selection with marker-assisted background selection, NILs of PB1121 and PB6 with high background recovery rates could be obtained after three backcrosses. Pu et al. [32] utilized MAS to obtain Chinese cabbage NILs resistant to clubroot disease (*CRb*) after three backcrosses.

*bls2* is a recessive BLS resistance gene, derived from Guangxi common wild rice accession DY19. To develop new *bls2*-resistant lines, this study used DY19 as the donor parent and 9311, a highly susceptible indica rice variety, as the recipient parent. After multiple generations of backcrossing and selfing, combined with MAS and inoculation identification, four new lines with significantly enhanced resistance to BLS were developed in the BC_5_F_4_ generation, with background recovery rates exceeding 98%. It demonstrates the effectiveness of MAS in constructing resistant NILs for recessive genes. MAS not only reduces the difficulty of screening homozygous recessive genes but also improves selection efficiency, accelerating the development of new resistant lines.

### 3.4. Identification of Selected Lines Using SNP Chip

SNPs represent the most abundant form of genetic variation in the plant genome [33]. The declining cost of sequencing technologies has significantly enhanced SNP detection efficiency through sequence alignment-based method [34]. SNP have become the most prevalent and reliable molecular marker for applications including high-throughput genotyping, variety authentication, bulked segregant analysis (BSA), and QTL mapping [35]. The 1K mGPS SNP chip can be used to detect genetic consistency between parents and offspring. This method can effectively identify whether there is contamination from foreign pollen or variety admixture in hybrid progeny. It can also be used for parent-offspring genotyping analysis by comparing genotypes of progeny with the parent at differentially homozygous loci. Yang et al. [36] analyzed the genetic diversity and structure of 317 fragrant glutinous rice germplasms from Guizhou, using 10 indica rice materials as controls, and identified 47 core germplasms using the 1K mGPS SNP chip. In this study, the 1K mGPS SNP chip was employed to verify the genetic consistency between the four resistant lines and their parents, and all showing a background recovery rate exceeding 98%. Notably, we confirmed that the insertion sites of introgressed segments in the four resistant lines correspond to the previously mapped regions of *bls2*. Using the SNP chip to detect the selected lines can not only precisely map the genomic segment containing the resistance gene but also conduct a genome-wide background analysis, establishing a solid foundation for precise breeding of disease-resistant varieties.

It was found by SNP chip detection that the *bls2* introgressed segments in all four resistant lines are relatively long, which may lead to the introduction of undesirable non-target traits. This likely resulted from using only positive selection markers without applying negative selection markers in both upstream and downstream regions of *bls2*. Studies have shown that relying solely on phenotypic selection and a limited number of molecular markers when introgressing the blast resistance gene *Pigm* may result in residual foreign segments spanning hundreds of kb or even Mb. However, high-density SNP (e.g., GSR40K chip) scanning can precisely define the boundaries of *Pigm*, retaining only the critical region (<100 kb) and significantly reducing linkage to irrelevant genes [37]. For the bacterial blight resistance gene *Xa21*, increasing SNP marker density in the target region allows more accurate identification of recombination events, thereby minimizing linkage drag. For example, screening for low-linkage SNP markers within a 500 kb upstream and downstream region of *Xa21* can effectively narrow the candidate interval and avoid the introduction of unfavorable alleles [37]. Therefore, during the resistant lines breeding process, we recommend implementing an integrated strategy incorporating MAS combining both positive and negative molecular markers, high-density SNP chip screening, multi-generation backcrossing, and phenotypic evaluation to mitigate linkage drag.

### 3.5. Application Value of bls2

In this study, the resistance reactions of the selected lines to four highly virulent *Xoc* strains from different sources were investigated during the seedling, tillering, and booting stages. There were highly significant differences in resistance to the same *Xoc* strain between the resistant lines and the recipient parent 9311. The resistant lines Y9-1, Y9-2, Y9-3, and Y9-4 demonstrated moderate or higher resistance levels to different strains across all growth stages, indicating that *bls2* possesses resistance against four *Xoc* strains throughout the entire growth period. The cloned dominant BLS resistance gene *Xo1* recognizes TALE proteins of *Xoc* and confers resistance to low-virulence strains from Africa and America, but this resistance is suppressed by iTALE [10]. Currently, most *Xoc* strains in Chinese fields carry two types of iTALE, rendering *Xo1* ineffective for practical application in China. Another resistance gene, *qBlsr5a*, does not confer resistance to strains carrying PthXo1-like TALEs [38]. Our research findings demonstrate that *bls2* provides resistance against four *Xoc* strains across all growth stages, making it an excellent genetic resource for breeding BLS-resistant varieties.

The resistant lines Y9-1, Y9-2, Y9-3, and Y9-4, despite carrying relatively long introgressed segments containing the resistance locus, showed no significant differences in agronomic traits such as plant height, effective panicle number, panicle length, seed setting rate, grain length, grain width, length-to-width ratio, and 1000-grain weight compared to the recipient parent 9311. This indicates that *bls2* is not linked to undesirable agronomic trait genes. These four resistant lines successfully retained the superior agronomic traits of the recipient parent while acquiring BLS resistance, providing valuable innovative germplasm for subsequent resistance breeding. Another cloned BLS resistance gene, *bls1*, also originated from Guangxi common wild rice [14]. However, the introduction of *bls1* into cultivated rice resulted in adverse effects such as yield reduction. There is still a lack of superior BLS resistance genes with both strong resistance and no linkage drag. Therefore, *bls2* is a rare gene that offers resistance against four *Xoc* strains across all growth stages and no linkage to undesirable traits, holding significant application value in BLS resistance breeding and rice production.

## 4. Materials and Methods

### 4.1. Experimental Materials and Breeding Methods

**Figure 5:**
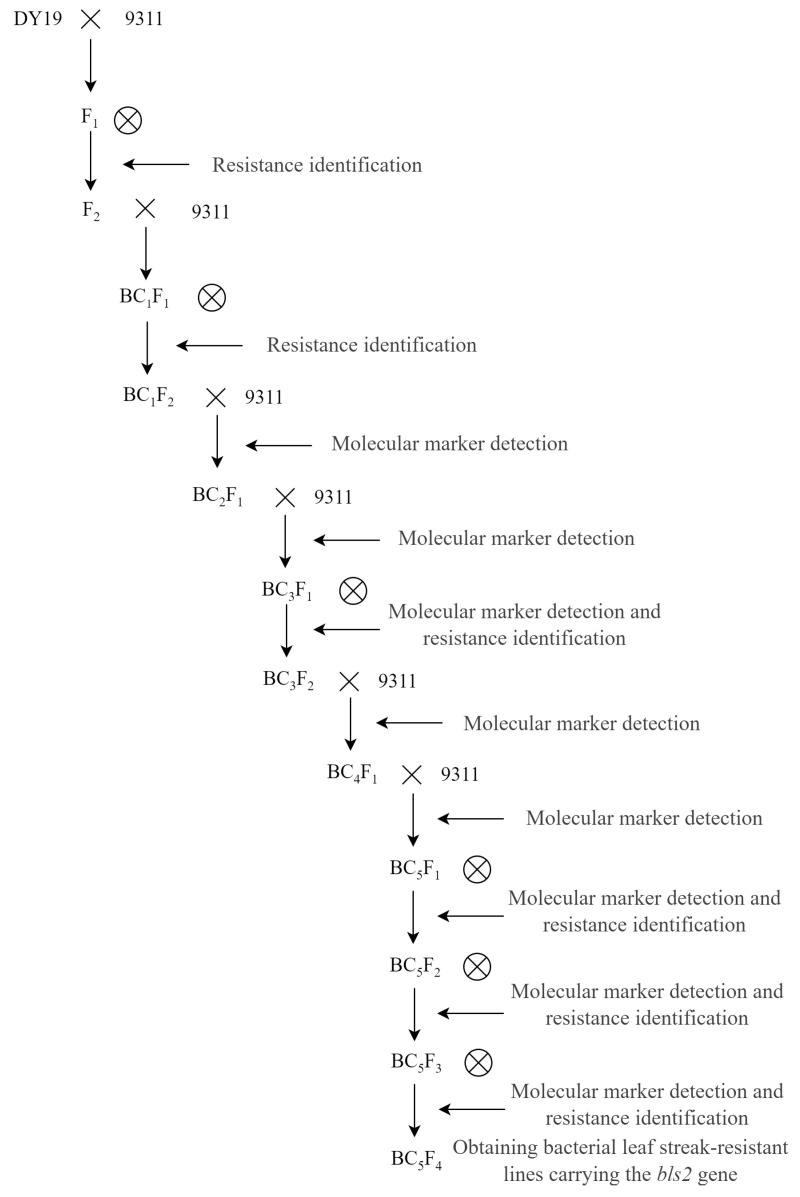
Flow chart of the creation of new lines carrying *bls2*.

DY19, a Guangxi common wild rice accession resistant to BLS, and 9311, a highly BLS-susceptible indica rice variety were used as experimental materials. The donor parent DY19 was crossed with the recipient parent 9311, followed by selfing. BLS-resistant F_2_ plants were selected through resistance inoculation identification and then backcrossed with 9311, followed by selfing to obtain the BC_1_F_2_ population. From this population, BLS-resistant plants were selected through resistance identification and backcrossed with 9311 again. Meanwhile, molecular markers obtained from *bls2* mapping were used to screen plants in each subsequent generation. After every two consecutive backcrosses, one selfing was performed, and resistance inoculation assays were conducted in each selfing generation. This process continued until the BC_5_F_2_ generation, where plants carrying the homozygous resistant marker genotypes with significantly enhanced BLS resistance and agronomic traits similar to those of 9311 were selected. These plants were then selfing twice to obtain stable lines. The process of developing the resistant lines is illustrated in Figure 5.

### 4.2. Molecular Marker

The three initially mapped primers (SL03, SL04, RM13592) were used. Based on the sequencing results of the two parents, four additional InDel primers were designed between SL03 and SL04 using the software Primer5.0 (PREMIER Biosoft International, Palo Alto, CA, USA). These seven primers were synthesized by Sangon Biotech (Shanghai) Co., Ltd., Shanghai, China (https://store.sangon.com/). The sequence information of the molecular markers is presented in Table 5.

### 4.3. DNA Extraction, PCR Amplification, and Electrophoresis Detection

The total genomic DNA of rice was extracted using the TIANGEN DNA Extraction Kit (TIANGEN, Beijing, China) according to the manufacturer’s instructions. The DNA was diluted to 20 ng/μL and used as a template for PCR amplification.

The PCR reaction system (10 μL) consisted of 1 μL DNA template (20 ng/μL), 1 μL primers (containing forward and reverse primers, 2 pmol/L each), 3 μL 2× Taq PCR Master Mix, and ddH_2_O to adjust the total volume to 10 μL. Amplification was performed using a PCR1000 DNA Thermal Cycler (Thermo, Waltham, MA, USA) under the following program: pre-denaturation at 95 °C for 5 min; 35 cycles of denaturation at 95 °C for 30 s, annealing at 55 °C for 30 s, and extension at 72 °C for 30~60 s; followed by a final extension at 72 °C for 5 min.

The PCR products were separated by 7% PAGE gel electrophoresis and visualized using silver staining.

### 4.4. SNP Chip Detection

Three plants were randomly selected from each line to extract DNA, and then the samples were sent to the Huazhi Biotech Co., Ltd., Changsha, China (https://www.higentec.com/) for DNA chip detection.

### 4.5. Bacterial Leaf Streak Resistance Identification

#### 4.5.1. Inoculation Strain

The *Xoc* strain used for gene fine mapping was the highly pathogenic Guangxi strain GX01, which was also the strain employed in the preliminary mapping of *bls2*. The strains used for resistant line identification included the highly virulent strains GX01 BNN3-*Xoc*, 2019-*Xoc* collected from Guangxi Province, South China, and YN-*Xoc* collected from Yunnan Province, Southwest China. All these strains were kindly provided by Professor Yongqiang He from the College of Agriculture at Guangxi University.

#### 4.5.2. Culture Method for Xoc Strain

*Xoc* strain was activated on NA medium at 28 °C for 48 h. A single colony was then selected and inoculated into 250 mL of NB medium, followed by expansion culture at 28 °C and 200 r/min for approximately 48 h until the logarithmic growth phase was reached. The culture was centrifuged at 5000× *g*/min for 5 min, the supernatant was discarded, and the bacterial pellet was collected. Sterile water was added to resuspend and dilute the bacteria to a concentration of 3 × 10^8^ CFU/mL for inoculation. To ensure that the inoculated bacteria were in the logarithmic growth phase, the bacterial suspension was prepared fresh for immediate use.

#### 4.5.3. Inoculation Method

The needle-puncture method was employed for inoculation assays. Two pins were fixed on a rubber pad with an interval of approximately 0.5 cm. A glass Petri dish and a sponge (about 2 cm thick) that could fit inside the dish were prepared. The Petri dish, the rubber pad with fixed pins, and the sponge were sterilized in advance. During inoculation, the bacterial suspension was poured into the Petri dish containing the sponge, allowing the sponge to fully absorb the suspension. Additional bacterial suspension was then added to fill two-thirds of the dish. The rice leaf was gently placed on the sponge in the Petri dish. At approximately 5 cm from the leaf tip, the two pins fixed on the rubber pad were used to prick the leaf on either side of the midrib. The rubber pad was then pressed gently against the sponge to ensure the pricked wound on the leaf fully contacted the bacterial suspension [39]. Two leaves per rice plant were inoculated, and the bacterial suspension in the Petri dish was replenished promptly during the inoculation process.

#### 4.5.4. Inoculation Treatment and Resistance Identification

The inoculation work is carried out at the seedling stage, tillering stage, and booting stage, respectively. For each rice material, rows 1–3 are inoculated with GX01, rows 5–7 with BNN3-*Xoc*, rows 9–11 with YN-*Xoc*, rows 13–15 with 2019-*Xoc*, and rows 17–19 with sterile water as the negative control, rows 4, 8, 12 and16 are left uninoculated and serve as spacer rows and backup plants.

Approximately 15 days after rice inoculation, lesion assessment was conducted. The length of four lesions per rice plant was measured using a vernier caliper. After comparing the four lesion lengths and excluding those with excessive variation, the average lesion length per plant was calculated to determine the resistance level. Referring to the resistance evaluation criteria established by Zhao et al. [40], the resistance level was classified based on lesion length. Lesions less than or equal to 1.5 cm were considered resistant, while lesions greater than 1.5 cm were classified as susceptible. Detailed resistance evaluation criteria are presented in Table 6.

#### 4.5.5. Rice Cultivation and Trait Investigation

The sowing dates, trial locations, and field management for the lines subjected to agronomic trait investigation were the same as those for the resistance identification trials. Rice was cultivated at the Scientific Research Experimental Base of the College of Agriculture, Guangxi University. The plants were transplanted individually in paddy fields, with six plants per row and a spacing of 15 cm × 25 cm between plants and rows. Field management followed standard practices for irrigation, fertilization, and pest control (excluding management for bacterial leaf streak). The agronomic traits of resistant lines from the BC_5_F_4_ generation were observed at maturity, using the recurrent parent 9311 as the control. Six plants from each line were measured, and key agronomic traits were recorded, including plant height, panicle length, number of panicles per plant, grain length, grain width, total grains per panicle, seed-setting rate, and 1000-grain weight.

#### 4.5.6. Data Statistical Analysis

The experimental data were analyzed using Student’s *t*-test to compare the differences in major agronomic traits between the resistant lines and 9311, and combined with analysis of variance (ANOVA) to compare the lesion lengths among rice materials after inoculation with different strains. Charts were generated using Excel, and statistical analysis was performed using IBM SPSS Statistics 26 (IBM, Armonk, NY, USA).

## 5. Conclusions

In this study, two molecular markers, ID2 and ID5, closely linked to BLS resistance gene *bls2*, were developed. Both markers demonstrated over 98% accuracy in detecting *bls2* and can be utilized for MAS. Four lines carrying the *bls2* gene developed in this study not only retained the BLS-resistance of Guangxi wild rice DY19 but also maintained the superior agronomic traits of cultivar 9311, providing valuable germplasm resources for breeding new BLS-resistant rice varieties. This demonstrates that continuous exploration of BLS-resistant germplasm resources, quantitative trait loci (QTLs), and key genes will effectively broaden the genetic basis for BLS-resistant rice breeding.

## Figures and Tables

**Table 1:** Annotation information of candidate genes.

Gene ID	Description
*Os02g0614966*	Leucine-rich repeat domain containing protein
*Os02g0615300*	LRR-receptor-like kinase (LRR-RLK) family protein
*Os02g0615400*	Leucine-rich repeat domain containing protein
*Os02g0615500*	LRR-receptor-like kinase (LRR-RLK) family protein
*Os02g0615800*	Leucine-rich repeat-receptor kinase-like protein
*Os02g0616100*	Similar to protein binding protein
*Os02g0616199*	Conserved hypothetical protein

**Table 2:** Accuracy of marker-assisted selection and field BLS-resistance identification.

Molecular Marker	Field BLS-Resistance Identification		Marker Assisted Selection		Subgroup Accuracy	Difference of Subgroup Accuracy	Group Accuracy
	Phenotype	Number of Plants	Number of Plants with Resistance Marker Genotypes	Number of Plants with Susceptible Marker Genotypes			
ID2	resistant	151	149	2	98.68	1.32	99.35
	Susceptible	156	0	156	100.00		
ID5	resistant	151	147	4	97.35	2.65	98.70
	Susceptible	156	0	156	100.00		
ID2/ID5	resistant	151	147	4	97.35	2.65	98.70
	Susceptible	156	0	156	100.00		

**Table 3:** The lesion lengths on the resistant lines infected by different *Xoc* strains at various growth stages.

Growth Stage	Material	*Xoc* Strain			
		GX01	BNN3-*Xoc*	YN-*Xoc*	2019-*Xoc*
Seedling stage	Y9-1	0.73 ± 0.060 (R) b	0.95 ± 0.143 (R) b	1.02 ± 0.190 (MR) b	1.03 ± 0.138 (MR) b
	Y9-2	0.77 ± 0.065 (R) b	0.81 ± 0.066 (R) b	0.96 ± 0.220 (R) b	0.99 ± 0.323 (R) b
	Y9-3	0.74 ± 0.084 (R) b	0.77 ± 0.079 (R) b	0.77 ± 0.094 (R) b	0.83 ± 0.133 (R) b
	Y9-4	0.72 ± 0.075 (R) b	0.91 ± 0.138 (R) b	1.01 ± 0.188 (MR) b	1.13 ± 0.134 (MR) b
	9311	3.03 ± 0.228 (HS) a	2.88 ± 0.213 (S) a	2.94 ± 0.183 (S) a	2.98 ± 0.428 (S) a
Tillering stage	Y9-1	0.83 ± 0.048 (R) b	1.00 ± 0.124 (MR) b	1.10 ± 0.191 (MR) b	1.20 ± 0.137 (MR) b
	Y9-2	0.74 ± 0.064 (R) b	0.80 ± 0.065 (R) b	0.80 ± 0.078 (R)c	0.84 ± 0.140 (R)c
	Y9-3	0.80 ± 0.057 (R) b	0.84 ± 0.063 (R) b	0.99± 0.219 (R) bc	1.04 ± 0.321 (MR) bc
	Y9-4	0.74 ± 0.048 (R) b	0.94 ± 0.132 (R) b	1.03 ± 0.175 (MR) bc	1.14 ± 0.133 (MR) bc
	9311	3.11 ± 0.418 (HS) a	3.01 ± 0.233 (HS) a	2.97 ± 0.200 (S) a	3.11 ± 0.131 (HS) a
Booting stage	Y9-1	0.69 ± 0.069 (R)c	0.91 ± 0.147 (R) b	0.99 ± 0.196 (R) b	1.09 ± 0.135 (MR) bc
	Y9-2	0.71 ± 0.075 (R)c	0.74 ± 0.079 (R) b	0.74 ± 0.089 (R) b	0.80 ± 0.139 (R)c
	Y9-3	0.74 ± 0.071 (R) bc	0.78 ± 0.059 (R) b	0.93 ± 0.224 (R) b	0.96 ± 0.315 (R) bc
	Y9-4	0.89 ± 0.116 (R) b	0.88 ± 0.139 (R) b	0.98 ± 0.183 (R) b	1.26 ± 0.132 (MR) b
	9311	2.84 ± 0.228 (S) a	2.68 ± 0.213 (S) a	2.74 ± 0.210 (S) a	2.78 ± 0.456 (S) a

The uppercase letters in parentheses represent the type of resistance (R: resistance, MR: moderate resistance, S: susceptibility, HS: High susceptibility). Data represent means ± SD, n = 16. Different lowercase letters indicate statistically significant differences among the rice materials (*p* < 0.05, ANOVA, Dunn’s post-hoc test).

**Table 4:** Agronomic traits in the resistant lines.

Material	Plant Height (cm)	No. of Effective Panicle	Spike Length (cm)	Setting Rate (%)
Y9-1	113.50 ± 2.83 ns	8.33 ± 1.53 ns	23.57 ± 2.99 ns	89.12 ± 2.13 ns
Y9-2	109.90 ± 4.88 ns	9.67 ± 1.53 ns	25.30 ± 1.74 ns	85.21 ± 3.71 ns
Y9-3	113.77 ± 5.41 ns	8.00 ± 1.00 ns	24.36 ± 0.41 ns	78.25 ± 4.89 ns
Y9-4	110.67 ± 5.82 ns	8.00 ± 2.65 ns	24.59 ± 1.83 ns	83.27 ± 2.39 ns
9311	111.43 ± 2.67	6.67 ± 0.58	24.44 ± 0.63	79.85 ± 6.45
**Material**	**Grain Length (cm)**	**Grain Width (cm)**	**Length-Width Ratio**	**1000-Grain Weight (g)**
Y9-1	1.21 ± 0.115 ns	0.28 ± 0.008 ns	4.37 ± 0.30 ns	28.33 ± 0.36 ns
Y9-2	1.47 ± 0.253 ns	0.29 ± 0.016 ns	5.09 ± 0.81 ns	27.62 ± 1.57 ns
Y9-3	1.51 ± 0.235 ns	0.29 ± 0.016 ns	5.28 ± 0.68 ns	26.29 ± 1.23 ns
Y9-4	1.29 ± 0.238 ns	0.28 ± 0.009 ns	4.64 ± 0.73 ns	27.59 ± 0.64 ns
9311	1.38 ± 0.140	0.29 ± 0.005	4.82 ± 0.43	27.79 ± 1.10

Data represent means ± SD, n = 6, ns represent no significance between the resistant lines and 9311 (*p* < 0.05, Student’s *t*-test).

**Table 5:** Nucleotide sequences of the primers.

Primers Name	Forward (5′–3′)	Reverse (5′–3′)
SL03	GCAAATTCCTCTGTTCTGTG	ACTTGCAAAGCAAATTCTGT
ID1	CATGCTGATGCGATTAAGACTGC	CTAACCTGTGCCTTGTTTGATGG
RM13592	GTGTCTGCATTTCTGTATGTGTGG	CGCTTAGCATTTACACACTCTCTCG
ID2	AGTGTCATCTACTCATCTTG	TTGTAACTCAGAGTGAACTA
ID5	GAGTCGGTATAGGCCCAGAG	TCCATTTATGCAGCTGTTCG
ID6	GTTGCATTTGATTTTCTACG	GCTTATGCCATTAAACTATG
SL04	TAAAAGTGGAGTCATCCGTC	GAGAATGGGTTGTGGATTAG

**Table 6:** Evaluation criteria for rice bacterial leaf streak resistance levels.

Lesion Length/cm	Disease Index	Resistant Type
0	0	Immunity
0.1–0.5	1	High resistance
0.6–1.0	3	Resistance
1.0–1.5	5	Moderate resistance
1.6–3.0	7	Susceptibility
>3.0	9	High susceptibilty

## Data Availability

All data generated or analyzed during this study are included in this published article and its Supplementary Tables.

## Supplementary Materials

The following supporting information can be downloaded at https://www.mdpi.com/article/10.3390/ijms26115264/s1.

## Abbreviations

*Xanthimonas oryzae* pv. *oryzicola* (*Xoc*); Bacterial leaf streak (BLS); Transcriptional ac-tivator-like effector (TALE); molecular marker-assisted selection (MAS).
